# Influence of the rod photoresponse on light adaptation and circadian rhythmicity in the cone ERG

**Published:** 2009-10-30

**Authors:** Morven A. Cameron, Robert J. Lucas

**Affiliations:** University of Manchester, Faculty of Life Sciences, Manchester, UK

## Abstract

**Purpose:**

In the mammalian retina, rod and cone pathways are fundamentally intertwined, with signals from both converging on cone bipolar cells to reach retinal ganglion cells. Psychophysical and electrophysiological data suggests that, as a consequence, rod signal transduction has a suppressive effect on the activity of cone pathways. It therefore might be assumed that the balance between rod and cone input to cone bipolar cells would be subject to dynamic regulation. There is evidence of light and time-of-day dependent alterations in this parameter. Here we set out to determine the extent to which such changes in rod-cone pathway convergence explain alterations in cone pathway function associated with light adaptation and circadian phase by recording cone electroretinograms (ERGs) in mice deficient in rod phototransduction.

**Methods:**

Cone-isolated ERGs elicited by bright flashes superimposed on a rod saturating background light were recorded from wild-type and rod transducin deficient (*Gnat1^−/−^*) mice. The process of light adaptation was observed by tracing changes in the ERG waveform over 20 min exposure to the background light in these genotypes, and circadian control by comparing responses at subjective midday and midnight.

**Results:**

The cone ERG b-wave exhibited significantly enhanced amplitude and reduced latency (implicit time) in *Gnat1^−/−^* mice under all conditions. Light adaptation was associated with a robust increase in b-wave amplitude in *Gnat1^−/−^* mice but, in contrast to wild types, almost no change in implicit time. *Gnat1^−/−^* mice retained circadian rhythms in the cone ERG with b-wave amplitudes larger and latencies reduced during the subjective day.

**Conclusions:**

Rod phototransduction has a strong suppressive effect on the cone ERG. Light adaptation in cone pathways relies in part on reductions in this effect, although mechanisms intrinsic to cone pathways also play an important role. Similarly, while changes in coupling between rod and cone pathways over the course of the day may contribute to circadian regulation of the cone pathway they are not sufficient to explain circadian rhythms in the wild-type cone ERG.

## Introduction

The retinal pathways conveying signals from rod and cone photoreceptors are fundamentally intertwined. Signals originating from both cones and rods must travel through cone bipolar cells to reach retinal ganglion cells [1-3]. Thus, although a distinction is often made between rod and cone bipolar cells, cone bipolar cells are, in fact, critical conduits of the rod signal. There are two known routes by which rod signals are delivered to ON cone bipolar cells. In the first, rod signals are conveyed initially through rod bipolar cells to AII amacrine cells, which make gap junction connections to ON cone bipolar cells. In the second, gap junction connections between rods and cones allow rod signals to bypass rod bipolar cells and employ the cone to cone-bipolar synapse [4].

The presence of rod signals in cone ON bipolar cells has the potential disadvantage of reducing their ability to respond to changes in cone activation under rod-saturating, photopic, conditions. It is perhaps then not surprising that the degree of coupling between rod and cone pathways is reduced under conditions favoring cone-based vision. First, light adaptation reduces the influence of rods on cone-based vision [5-8]. This probably arises in part through decreased coupling among AII amacrine cells and between AII amacrine cells and cone ON bipolar cells associated with light-induced release of dopamine and nitric oxide [9,10]. Second, a circadian rhythm in the degree of gap junction coupling between rods and cones has been reported, with greatly reduced coupling observed during the subjective day [11].

The effects of both circadian regulation and light adaptation on the activity of cone pathways can be readily observed using the electroretinogram (ERG). Many studies examining the cone ERG have shown increases in amplitude and decreases in latency of the b-wave (representing the activity of cone ON bipolar cells) during light adaptation [12-15]. Similarly, circadian variation in the ERG has been reported [16-18]. We recently showed in the mouse that the cone b-wave elicited by a flash superimposed on a rod saturating background light showed reduced amplitude and increased latency in the subjective night [19,20].

Here, we set out to assess the extent to which modulation of the mouse cone ERG according to light adaptation and circadian phase could be explained by changes in the degree of coupling between rod and cone pathways. Our approach was to employ mice lacking the α-subunit of rod transducin (*Gnat1^−/−^*). Rod transducin is considered a critical element in the rod phototransduction cascade, and *Gnat1^−/−^* mice have been used as a model of rod inactivation without retinal degeneration [21]. They therefore provide an opportunity to record the cone ERG in the absence of the tonic rod signal that would ordinarily be present under photopic conditions.

We find that the cone ERG of *Gnat1^−/−^* mice had increased amplitude and decreased latency compared to wild types, confirming the strong suppressive effect of tonic rod signals on cone pathways. Our data are consistent with changes in coupling between rod and cone pathways being a major mechanism of light adaptation. However, *Gnat1^−/−^* cone ERGs retained significant light adaptation and circadian rhythmicity, indicating that events intrinsic to the cone pathway are also important components of these processes.

## Methods

### Animals

Mice were bred and housed in the University of Manchester. All procedures were conducted according to the UK Animals (Scientific Procedures) Act of 1986. *Gnat1^−/−^* mice were from a C57BL/6 and 129sv mixed strain background, and C57BL/6 wild types were used as controls. For over a week before ERG, mice (70–120 days of age) were housed under a stable 12 h:12 h light-dark cycle (white fluorescent ~0.7 log W/m^2^ at cage floor). They were allowed food and water ad libitum.

### Electroretinography

Anesthesia was initiated with 70 mg/kg ketamine and 7 mg/kg xylazine and maintained with 72 mg/kg ketamine and 5 mg/kg xylazine. Mydriatics, 1% tropicamide and 2.5% phenylephrine, and 0.5% hypromellose solution (Alcon, Puurs, Belgium) were applied to the recording eye before application of a contact lens-type electrode [22]. A needle reference electrode (Ambu®; Neuroline, Ølstykke, Denmark) was inserted approximately 5 mm from the base of contralateral eye, distal enough to preclude signal contamination. A silver wire bite bar was used to support the head and to act as ground. Electrode setup and injection of anesthetic agents was conducted under dim red light (<0.1 W/m^2^; >650 nm). Electrodes were connected via a signal conditioner (Model 1902 Mark III; CED, Cambridge, UK; signal differentially amplified, ×3000; and band-pass filtered 0.5 to 200 Hz), and digitized (Model 1401, CED) to a Windows PC (sampling rate 2 kHz) running the Signal 2.16 Software (CED). Core body temperature was maintained by placing animals in a custom-made hose coil connected to a constant temperature water source. In all cases electrode stability was confirmed over at least 10 min before recording.

Dark-adapted irradiance responses were elicited by white flash stimuli from a xenon arc source (Cairn Research Ltd., Faversham, UK) reflected in a custom-made Ganzfeld dome and attenuated with neutral density filters (Edmund Optics, York, UK) as required to achieve corneal irradiances in the range –8.45 to 0.05 log W/m^2^ (roughly –5.5 to 2.5 log cd-sec/m^2^). An electrically controlled, mechanical shutter (Cairn Research Ltd.) was used to apply a series of single 15-ms flashes, each starting 200 ms after sweep onset. Interstimulus interval was 1.5 s at the dimmest intensities and was increased, proportionally with irradiance, to 30 s at the brightest intensities. Similarly, the number of repetitions decreased from 30 to 6 as the stimulus intensity was increased. The b-wave amplitude was quantified by summing the absolute a-wave amplitude (where present) and the b-wave amplitude when filtered (low pass 5 Hz) to remove the influence of oscillatory potentials. Implicit times were measured from the stimulus onset to peak of the b-wave.

Cone-driven activity was isolated by measuring responses to a series of bright white flashes (Grass Model PS33 Photic Stimulator; Astro-Med, Inc., West Warwick RI, fitted with a 400 nm high pass filter; 10 µs duration; peak corneal irradiance 1 log W/m^2^, which is approximately 6 log cd-sec/m^2^) applied at a frequency of 0.75 Hz against a uniform white background light (metal halide source) of sufficient intensity (0.7 W/m^2^, which is roughly 5.7 log cd-sec/m^2^) to saturate rods, but not cones [23]. The background light was left on over the following 20 min and photopic ERGs recorded continuously at 0.75 Hz. The peak of the b-wave was identified from these averaged traces and its amplitude (measured from the stable baseline immediately before stimulus onset) and implicit time (from start of flash) calculated. Averaged waveforms were obtained for every 25 frames then filtered (low pass 5 Hz) to exclude oscillatory potentials.

Responses were recorded at subjective midday circadian time 6 (CT6) and subjective midnight (CT18), after 18 and 30 h dark adaptation, respectively. The process of light adaptation for all parameters was fitted with a one phase decay exponential function (Equation 1) by GraphPad Prism software:

Equation 1:

$$Y=(Y_{0}-a)e^{-kx}+a$$

where *Y_0_* represents the value of Y when X=0, *a* the upper asymptote and *k* the rate constant. Irradiance response curves were fitted with either a singular sigmoidal dose response curve (Equation 2) in the case of implicit time,

Equation 2:

$$Y=b+\frac{\left(a-b\right)}{1+10^{(LogEC50-x)h}}$$

or a biphasic dose response curve (combining two sigmoidal functions covering distinct irradiance ranges; Equation 3)

Equation 3:

$$Y=b+\frac{(a-b)k}{1+10^{(LogEC501-x)h1}}+\frac{\left(a-b)(1-k\right)}{1+10^{(LogEC502-x)h2}}$$

in the case of amplitude. In these equations, *a* represents the upper saturating asymptote, *b* the lower asymptote, *h* the hill slope, and *k* the proportion of maximal response due to the more potent phase for the biphasic curve. Light intensity was expressed on a log scale as log W/m^2^ for irradiance response curves.

## Results

### Dark-adapted ERGs from *Gnat1^−/−^* mice show cone-like characteristics

**Figure 1:**
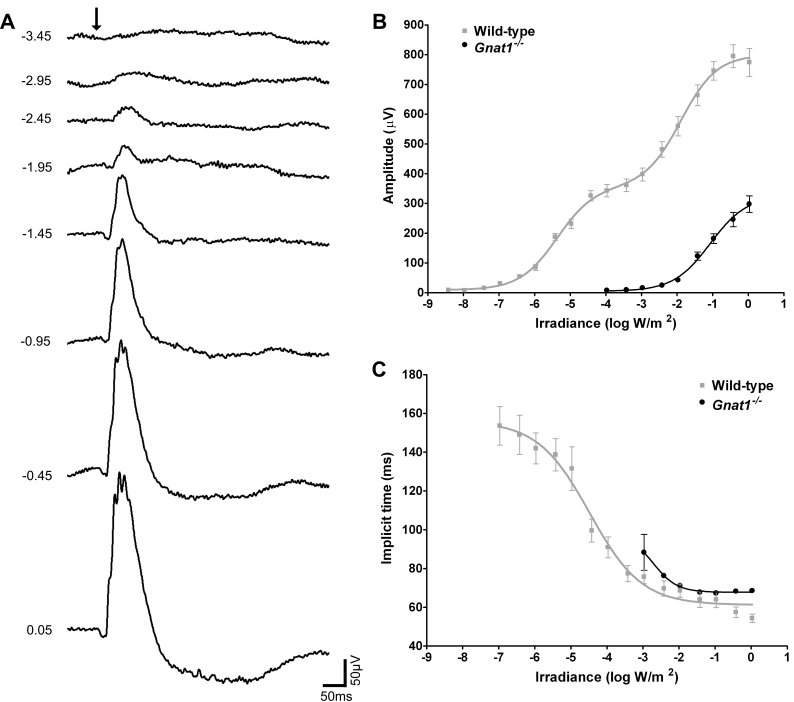
Dark-adapted ERG of *Gnat1^−/−^* mice. **A** shows representative dark-adapted ERG traces from *Gnat1^−/−^* mice recorded in response to white flashes of increasing irradiance at subjective midday (CT6) after approximately 18 h dark adaptation. **B** and **C** show quantification of b-wave amplitude and implicit time of ERGs from *Gnat1^−/−^* (black) and wild-type (gray) mice. Implicit time could only be measured when a reliable b-wave was discerned and is therefore absent at lower flash intensities. The arrow in **A** indicates flash onset and numbers on left indicate irradiance in log W/m^2^ (these radiometric irradiances equate to ~–1.0 to 2.5 log cd-sec/m^2^). Data in **B** and **C** is shown as mean±SEM. Replicates for **B** and **C** are: n=6 for *Gnat1^−/−^*; n=8 wild-type.

Flashes presented against a dark background at subjective midday (CT6) elicited ERGs from *Gnat1^−/−^* mice with a waveform similar to that of a classical cone ERG, comprising a large b-wave and very small a-wave (Figure 1A). In agreement with previously published data, this dark-adapted ERG showed a marked (roughly 4 log unit) reduction in sensitivity compared with wild types (Figure 1B). Moreover, even at the highest flash intensity neither b-wave amplitude nor implicit time was equivalent to that of wild types. These data are in agreement with previously published data that *Gnat1^−/−^* mice retain cone-based but not rod-based vision [21].

### Cone ERGs are substantially altered in Gnat1^−/−^ mice

**Figure 2:**
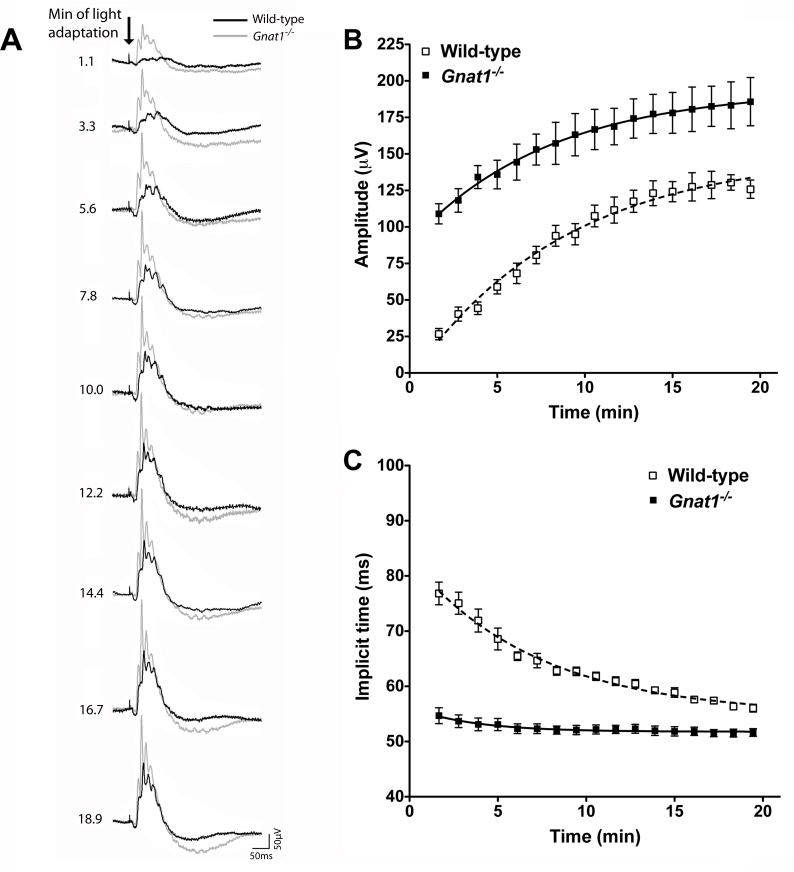
Comparison of cone ERG traces from *Gnat1^−/−^* and wild-type mice over the course of light adaptation. **A** shows representative ERG traces from *Gnat1^−/−^* (gray trace) and wild-type (black trace) mice recorded at CT6 in response to a bright white flash superimposed on a rod saturating background. At the beginning of light adaptation (top), b-waves were much more noticeable in *Gnat1^−/−^* animals than in wild-types. The magnitude of this difference was reduced as light adaptation proceeded. The arrow in **A** depicts flash onset, scale bar=50 ms (x-axis), 50 μV (y-axis) and numbers to left correspond to the time exposed to the rod saturating background in minutes. **B** shows quantification of b-wave amplitude against time spent exposed to the adapting light in *Gnat1^−/−^* (closed squares) compared to wild-type (open squares). *Gnat1^−/−^* mice show significantly larger responses than wild-types (F test curve fit comparison p<0.0001). **C** shows that b-wave implicit time (IT) was much reduced in *Gnat1^−/−^* mice compared to wild-types across all time points (F test curve fit comparison p<0.0001). Data in **B** and **C** is shown as mean±SEM. Replicates for **B** and **C** are: n=10 for wild-types, n=8 for *Gnat1^−/−^*. All data mean±SEM; n=9 for wild-types, n=8 for *Gnat1^−/−^*. The estimated parameters for curve fit for the curves in **B** (wild-type) are, amplitude: Y_0_=-5.061 a=149, k=0.124, implicit time: Y_0_=83.39, a=54.94, k=0.143. The curve fit for **C** (*Gnat1^−/−^*) estimated parameters are, amplitude: Y_0_=88.72, a=194.4, k=0.127, implicit time: Y_0_=56.23, a=51.78, k=0.283.

There were substantial differences in the cone isolated ERG waveform of wild type and *Gnat1^−/−^* mice recorded at CT6 (Figure 2A). This was particularly apparent in the first minute of exposure to the rod saturating background light, when the cone ERG is all but absent in wild types, but very noticeable in *Gnat1^−/−^* mice. This suggests that the tonic rod signal has a large suppressive effect on the amplitude and implicit time of the cone ERG b-wave.

Over the ensuing 20 min of light adaptation, the wild-type trace showed an increase in b-wave amplitude and a reduction in its implicit time (Figure 2). Similar changes in amplitude and implicit time were also observed in the *Gnat1^−/−^* b-wave over this timeframe (two-way ANOVA; p<0.0001), confirming that this genotype retains the ability to show light adaptation (Figure 2B,C). Nonetheless, throughout the recording period, the *Gnat1^−/−^* b-wave had enhanced amplitude and reduced implicit time compared to wild types (two-way ANOVA; p<0.0001 for both amplitude and implicit time).

Although *Gnat1^−/−^* mice showed light adaptation in both b-wave amplitude and implicit time, the magnitude of these effects was significantly reduced compared to wild types. The b-wave amplitude in *Gnat1^−/−^* mice increased by roughly 70 μV over 20 min of light adaptation as opposed to roughly 90 μV in wild-type mice. Nonetheless, there was little convergence in this parameter between genotypes over the course of adaptation, and curve fits for the data had divergent saturating asymptotes. This implies that differences in b-wave amplitude would be retained even under very extended light adaptation.

The magnitude of light adaptation in b-wave implicit time was substantially reduced in *Gnat1^−/−^* mice. Indeed, it appeared to be restricted to a modest (approximately 5 ms) decrease in latency over the first 2–3 min of exposure to the background light. Unlike amplitude, b-wave implicit time in the two genotypes appeared to converge over the course of adaptation, suggesting that under extended light adaptation they may have become indistinguishable.

### Circadian rhythms in the wild-type cone ERG

**Figure 3:**
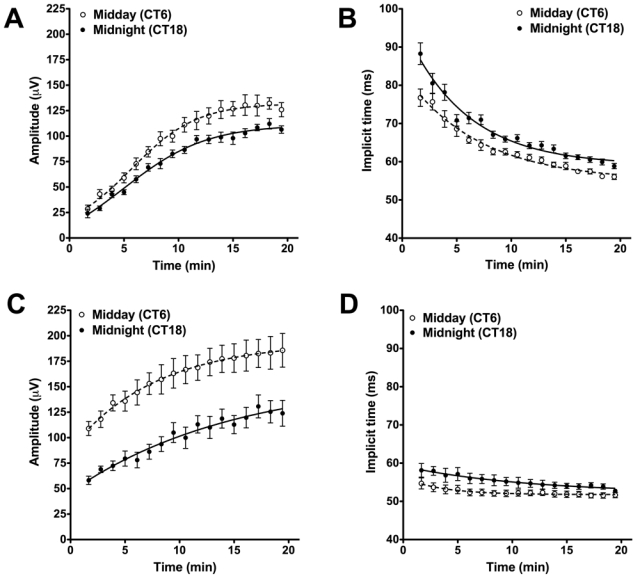
Circadian rhythms in the wild-type and *Gnat1^−/−^* cone ERG. Wild-type mice showed significant difference in b-wave amplitude (**A**) and implicit time (**B**) over the course of light adaptation when measured at CT6 (open circles) compared to CT18 (closed circles) F test curve fit comparison p<0.0001 for each parameter. Similar effects of circadian phase were observed on b-wave amplitude (**C**; F test p<0.0001) and implicit time (**D**; F test p<0.0001) in *Gnat1^−/−^* mice. All data are shown as mean±SEM. Replicates for **A** and **B** were: n=10 (CT6), n=8 (CT18), for **C** and **D**: n=8 (CT6), n=6 (CT18). The estimated parameters for curve fit for the curves in **A** were, CT6: Y_0_=-5.061 a=149, k=0.124, CT18: Y_0_=-2.029 a=127, k=0.1082. Estimated parameters for **B**, CT6: Y_0_=83.39, a=54.94, k=0.143, CT18: Y_0_=96.08, a=59.07, k=0.176. Estimated parameters for **C**, CT6: Y_0_=88.72, a=194.4, k=0.127, CT18: Y_0_=46.62, a=159.3, k=0.0666. Estimated parameters for **D**, CT6: Y_0_=56.23, a=51.78, k=0.283, CT18: Y_0_=59.20, a=52.11, k=0.087.

As previously described, we saw circadian dependence to both the amplitude and implicit time of the wild-type cone b-wave (Figure 3A,B). At both subjective midday (CT6) and midnight (CT18), light adaptation induced changes in both b-wave amplitude and implicit time (two-way ANOVA; p<0.0001 for both), but even accounting for this effect, b-wave amplitude was larger and latency reduced at CT6 versus CT18 (two-way ANOVA; p<0.05 for amplitude, p<0.01 for implicit time).

### Circadian rhythms in the cone ERG of Gnat1^−/−^ mice

To determine whether circadian control of coupling between rod and cone pathways could explain the rhythms in cone b-wave amplitude and implicit time we observe, we next assessed rhythmicity in the *Gnat1^−/−^* cone ERG. Comparison of the b-waves at CT6 and CT18 revealed the retention of a robust circadian rhythm in these animals (Figure 3C,D). As with the wild-type mice, the b-wave amplitude was significantly larger at CT6 than CT18 throughout light adaptation (Figure 3A, two-way ANOVA, p<0.01). There was also a significant circadian rhythm in the implicit time, with reduced latency responses at CT6 (Figure 3B, two-way ANOVA, p<0.05).

## Discussion

The *Gnat1^−/−^* mouse provides a unique opportunity to determine what happens to cone signals in the retina in the absence of rod phototransduction. It employs a genetic manipulation whose primary effect on rod phototransduction [21] is well understood, highly specific, and should be consistent between individuals and stable across time. Furthermore, while *Gnat1^−/−^* mice suffer slight retinal degeneration [21], its magnitude is much less than in other models of inherited rod dysfunction [24-26].

Under all conditions, the *Gnat1^−/−^* cone ERG b-wave had consistently enhanced amplitude and reduced implicit time compared to wild-type controls. It is possible that unexpected secondary effects of *Gnat1*-loss on retinal wiring (e.g., modest rod photoreceptor loss could reduce the leak of cone signals into neighboring rods through gap junctions) could contribute to these effects. Strain differences could also be a consideration as *Gnat1^−/−^* mice have mixed C57BL/6:Sv129 background, although this seems unlikely as, for the known QTL, lower amplitude is coupled to reduced latency [27]. The more interesting possibility is that these findings reflect an inhibitory influence of rod phototransduction on cone signals in the retina, a suggestion consistent with evidence that cone ERGS are similarly enhanced in rod opsin knockout mice [26]. Based upon what is known of retinal circuitry, the most likely mechanistic origin for such an effect is suppression of cone-dependent flash responses by appearance of a constitutive rod signal driven by the background light in cone ON bipolar cells.

The difference between *Gnat1^−/−^* and wild-type ERGs was particularly marked immediately after the rod saturating background light was switched on. At this time the wild-type cone ERG was practically absent, while *Gnat1^−/−^* mice retained a strong response. This supports the suggestion that such rod-dependent suppression of cone pathway responses is particularly prevalent when the retina is dark-adapted and is gradually reduced during light adaptation [6,7,14,15,28]. A likely source for such an effect is modulation of gap junction coupling between rod and cone pathways.

Gap junctions link rod and cone pathways by connecting neighboring rods to cones and AII amacrine cells to ON cone bipolar cells. Dopamine, and compounds that cause the release of second messengers like cGMP (such as nitric oxide) are released from amacrine cells in the inner retina in response to light and elicit closure of gap junctions between rods and cones, among neighboring AII amacrine cells, and between AII amacrine cells and ON cone bipolar cells [9,10,29]. The enhanced gap junction coupling within the retina in the dark should therefore ensure that rod signals provide a large contribution to the activity of the cone circuitry. This would mean that immediately following presentation of the adapting field, a strong tonic rod signal is routed through the cone circuitry. Thanks to response compression in elements of this circuitry, the incremental cone-dependent response to flash presentation may therefore be relatively small. The longer the retina is exposed to the adapting field, the more isolated from the cone circuitry the rod responses would become, causing cone responses above this adapting field to be more noticeable. The *Gnat1^−/−^* mice, however, lack this rod input, meaning that, even at the start of light adaptation, the cone circuitry conveys only cone signals, resulting in increased amplitude and reduced implicit time for the cone ERG b-wave. Since *Gnat1^−/−^* mice show almost no light adaptation in implicit time, this parameter may be almost exclusively determined by the degree of coupling between rod and cone pathways. However, *Gnat1^−/−^* mice still exhibit strong adaptation in b-wave amplitude. It remains possible that this reflects a change in coupling between rod and cone pathways insofar as the cone signal may, to some extent, be “diluted” in rod pathways, but it seems more likely that it originates with additional light adaptive mechanisms, such as those intrinsic to the cone photoreceptors [30].

Another factor known to determine the extent of gap junction coupling within the mammalian retina is the circadian clock. Ribelayga et al. [11]. showed greatly enhanced coupling within the population of rod and cone photoreceptors in the subjective night compared to the subjective day. This provides an attractive potential mechanism for the circadian rhythms in cone b-wave amplitude and implicit time that we, and others, have previously reported in mice [20,31]. Thus, enhanced flow of rod signals into cone ON bipolar cells at night could suppress the cone response under high background lights, resulting in reduced b-wave amplitude and increased latency. However, we show here that *Gnat1^−/−^* mice retain circadian rhythms in both b-wave amplitude and implicit time. Thus, while our data do not question the importance of circadian control of rod:cone coupling in regulating the retinal light response, they do show that this is not the only mechanism by which circadian clocks influence cone pathways.

Possibly the most surprising aspect of these results is that light adaptation in amplitude and implicit time of the cone b-wave are separable. Thus, *Gnat1^−/−^* mice show strong light adaptation in b-wave amplitude even as implicit time remains fairly constant. As both parameters have been previously shown to change in tandem [12-14,28], it had been assumed that they reflect the same adaptive mechanism. Our findings with *Gnat1^−/−^* mice indicate that this may not be the case. As the adaptation-dependent changes in cone b-wave amplitude are relatively large in this genotype, they must arise to a large extent via mechanisms intrinsic to the cone pathway (including perhaps in the cone photoreceptor itself). By comparison, light adaption in implicit time is almost lost in *Gnat1^−/−^* mice. We have previously shown that light-dependent dopamine release is impaired in this genotype and this could, in theory, explain the low amplitude of light adaptation in implicit time [32]. This, however, feels unlikely as light adaptation (and by implication dopamine) typically decreases implicit time, and this parameter is constitutively small in *Gnat1^−/−^* mice. Rather, a more direct influence of rod signals on response latency of the cone pathway is implied. The fact that rods exert such a strong influence on this parameter under relatively dark-adapted conditions could be functionally important in synchronizing rod and cone signal kinetics under mesopic conditions (when both photoreceptors convey visual information) to accurately resolve the visual scene.
